# Investigating the Effect of Emetic Compounds on Chemotaxis in *Dictyostelium* Identifies a Non-Sentient Model for Bitter and Hot Tastant Research

**DOI:** 10.1371/journal.pone.0024439

**Published:** 2011-09-08

**Authors:** Steven Robery, Janina Mukanowa, Nathalie Percie du Sert, Paul L. R. Andrews, Robin S. B. Williams

**Affiliations:** 1 School of Biological Sciences, Centre for Biomedical Sciences, Royal Holloway University of London, Egham, United Kingdom; 2 Division of Biomedical Sciences, St. George's University of London, London, United Kingdom; Cardiff University, United Kingdom

## Abstract

Novel chemical entities (NCEs) may be investigated for emetic liability in a range of unpleasant experiments involving retching, vomiting or conditioned taste aversion/food avoidance in sentient animals. We have used a range of compounds with known emetic /aversive properties to examine the possibility of using the social amoeba, *Dictyostelium discoideum*, for research into identifying and understanding emetic liability, and hence reduce adverse animal experimentation in this area. Twenty eight emetic or taste aversive compounds were employed to investigate the acute (10 min) effect of compounds on *Dictyostelium* cell behaviour (shape, speed and direction of movement) in a shallow chemotaxic gradient (Dunn chamber). Compound concentrations were chosen based on those previously reported to be emetic or aversive in in vivo studies and results were recorded and quantified by automated image analysis. *Dictyostelium* cell motility was rapidly and strongly inhibited by four structurally distinct tastants (three bitter tasting compounds - denatonium benzoate, quinine hydrochloride, phenylthiourea, and the pungent constituent of chilli peppers - capsaicin). In addition, stomach irritants (copper chloride and copper sulphate), and a phosphodiesterase IV inhibitor also rapidly blocked movement. A concentration-dependant relationship was established for five of these compounds, showing potency of inhibition as capsaicin (IC_50_ = 11.9±4.0 µM) > quinine hydrochloride (IC_50_ = 44.3±6.8 µM) > denatonium benzoate (IC_50_ = 129±4 µM) > phenylthiourea (IC_50_ = 366±5 µM) > copper sulphate (IC_50_ = 1433±3 µM). In contrast, 21 compounds within the cytotoxic and receptor agonist/antagonist classes did not affect cell behaviour. Further analysis of bitter and pungent compounds showed that the effect on cell behaviour was reversible and not cytotoxic, suggesting an uncharacterised molecular mechanism of action for these compounds. These results therefore demonstrate that *Dictyostelium* has potential as a non-sentient model in the analysis of the molecular effects of tastants, although it has limited utility in identification of emetic agents in general.

## Introduction

Emetic research employs a range of animal models, either to identify the emetic liability of a novel chemical entity (NCE) or to characterise mechanisms giving rise to emesis [1]. Common models can be divided into those that have the ability to vomit (e.g. ferret, house musk shrew, dog and cat), and those that lack the emetic reflex (e.g. rats and mice) [2]. In rats, pica, the ingestion of a non-nutritive substance such as kaolin, and conditioned taste aversion/food avoidance (CTA/CFA) are used as an emetic-like readout [3]. Considerable variability in the sensitivity to emetic compounds exists between animal models, due to the multiple pathways available for induction of the reflex, and differences in receptor pharmacology and distribution, and metabolic pathway regulation [4], [5]. This variability therefore makes it difficult to establish a single animal model for emetic research, and encourages a multi-model approach and increasing animal usage [5].

The very nature of emetic research has the potential to cause considerable distress in the subjects, and some emetic compounds (e.g. cisplatin) induce intense retching and vomiting and a protracted emetic response that can last for several days [6]. To reduce the number of animals needed for these adverse tests, Holmes et al. [5] suggested a tiered approach to identify potential emetic liability of NCEs early in compound optimisation. In this approach, a series of individual assays would be performed in order to reduce the final number of compounds tested on sentient models. The first tier in this approach would involve the *in silico* analysis of novel compounds under investigation, whereby structures of known emetic efficacy are compared with novel compounds under investigation employing previously recorded data from *in vivo* studies. Secondly, a simple non-sentient model would be used to screen for compounds showing strong effects associated with other emetic compounds. Thirdly, tissue culture experiments would be employed using mammalian cell lines to predict emetic liability, and then finally animal models would be incorporated. This approach could substantially reduce the number of animal experiments by excluding many compounds with potential emetic liability at an earlier stage. This tiered approach requires development of a simple non-sentient model system capable of identifying emetic liability of compounds in a high-throughput type screen.

*Dictyostelium* is a simple model system, widely used in the analysis of cell signalling, development, and cell behaviour during movement [7]–[10]. The genome of the model has been sequenced [11], identifying a wide range of homologues related to human disease proteins and associated intracellular signalling pathways. Many of these proteins and related pathways are absent in other simple model systems such as *Saccharomyces cerevisiae* and *Schizosaccharomyces pombe* [11], [12], suggesting *Dictyostelium* may have specific advantages over other commonly used non-sentient models.

*Dictyostelium* is increasingly being used in biomedical research [12]–[14], in for example, the analysis of mitochondrial disease [15], in Alzheimer's disease signaling [16], and in understanding pathways of microbial infection [17]. In many of these studies, and in other more pharmacologically-oriented projects, *Dictyostelium* has been used to analyse drug-induced changes in behaviour during movement at a cellular level [18]. For example, in the analysis of bipolar disorder drugs valproic acid and lithium [19], [20], for potential chemotherapy research [13], [21], [22], and for the vasodilator nitric oxide [23].

In this paper, we explore the utility of using *Dictyostelium* as a simple non-sentient model in the tiered approach to reducing animals in testing for emetic liability, as proposed by Holmes et al. [5]. This was investigated by monitoring cell behaviour (speed, shape and direction of movement) following exposure to a range of compounds known to induce emesis, pica or CTA/CFA. The broad categories of compounds investigated (summarised in Table 1) include: tastants (bitter and pungent [“hot”] compounds), cytotoxic anti-cancer agents, selective receptor agonists and antagonists and metal salts. Our results indicate that tastants (both bitter and hot compounds) cause a rapid, pronounced and concentration-dependent effect on cell behaviour, although a range of cytotoxic and receptor agonist/antagonists compounds giving rise to emetic or taste aversive responses had no effect. These results suggest that *Dictyostelium* may provide a new model for the analysis of bitter and hot compound perception and signalling, although it shows little functionality as a generalised predictor of emetic function for novel chemical entities.

**Table 1:** Emetic or taste aversive compounds assessed for effects on *Dictyostelium* behaviour during chemotaxis.

Generic Target	Target Receptor/ Mechanism of Action	Common Name	Concentration	Effect on *Dictyostelium*	Species	Dose Range	Reference
Receptor Agonist/ Tastant	T2R Receptor ligand	Denatonium Benzoate	0.05–10 mM	Y	R*, H*	0.01–10 mM	[36], [39], [63], [72]
		Phenylthiourea	0.05–5 mM	Y	H*	2–5 mM	[63]
		Quinine HCl	0.05–1 mM	Y	R*	0.0082–250 mM	[36], [73]
	TRPV1 Receptor Agonist	Capsaicin	0.01–0.3 mM	Y	S	0.04–0.4 mg/kg	[28], [74]
		Resiniferatoxin	1 µM, 10 µM	N	S	0.1–1000 µg/kg	[27], [29], [75], [76]
Cytotoxic	Cytotoxic/DNA Damage??	5-Fluorouracil	250 µM	N	F,R,S,H	35–100 mg/kg	[77]–[79]
		Actinomycin D	10 µM, 700 µM	N	D,R	0.13–0.25 mg/kg	[79], [80]
		Cisplatin	50 µM, 300 µM	N	F,D,R,S, H	3–20 mg/kg	[6], [29], [80]–[87]
		Cycloheximide	5 mM	N	F,D	20 mg/kg	[81]
		Methotrexate	50 µM, 250 µM	N	S,H	80 mg/kg	[77], [78]
		Streptozotocin	1 µM	N	H	14–27 mg/kg	[88]–[90]
		Vincristine	1 µM	N	R	0.1–1 mg/kg	[79]
ReceptorAntagonist	Extracellular Enzyme Inhibitor	Digoxin	1 µM	N	C,H	0.2–0.6 mg/kg	[91], [92]
	PDEIV Inhibitor	Rolipram	10 µM, 700 µM	Y	F,D,R,S	0.5–10 mg/kg	[60], [93]–[95]
	SSRI/Transmitter Uptake Inhibitor	Fluoxetine	6.5 µM	N	S	60 mg/kg	[96]
Receptor Agonist	5-HT Receptor Agonist	5-hydroxytryptamine	1 µM, 100 µM	N	S	4–10 mg/kg	[96]–[98]
	Dopamine Receptor Agonist	Apomorphine HCl	10 µM, 1 mM	N	F,D,R,H	0.1–10 mg/kg	[80], [81], [83], [85], [99]–[101]
	Ligand Gated Ion Channel Activator	Veratridine HCl	30 µM, 500 µM	N	D, C	0.02–0.25 mg/kg	[102]–[104]
	Neurokinin Receptor Agonist	Substance P	1 µM	N	D	0.03–0.2 mg/kg	[105]
	Nicotinic Receptor Agonist	Nicotine	6 µM, 100 µM	N	F,D,R,S,H	1.5–20 mg/kg	[29], [75], [87], [97], [106]–[109]
	Opioid Receptor Agonist	Loperamide HCl	1 µM, 100 µM	N	F	0.5 mg/kg	[85], [110], [111]
Other	CNS depressant	Lithium Chloride	10 mM	N	R,S,H	50–200 mg/kg	[109], [112], [113]
	Enteroendocrine Cell Stimulant	Metformin	500 µM, 10 mM	N	H*	1–30 µM	[114], [115]
	Free Radical Generator	Pyrogallol	500 µM, 10 mM	N	S	128 mg/kg	[27], [116]
	Gastric mucosal irritant	Copper Sulphate	0.16– 5 mM	Y	F,D,R,S, H	5–120 mg/kg	[29], [85], [97], [109], [117], [118]
		Copper Chloride	1.6 mM	Y			Based on concentratio of copper sulphate: [29], [85], [97], [109], [117], [118]
		Zinc Sulphate	1.6 mM	N	H	1%	[119]
	Prostaglandin	PGF_2α_	1 µM, 100 µM	N	F,S	1–13.5 mg/kg	[117], [120]

A range of emetic or taste aversive compounds within the categories of tastants, cytotoxic agents, generalised receptor agonists/antagonists, and other compounds were selected for acute exposure to chemotaxing *Dictyostelium* cells. These compounds have a range of target receptors and/or mechanisms of action, as indicated. Compound concentrations employed in these *Dictyostelium* experiments (shown here) were derived from the experimental dose range for each compound used in emetic-related experiments in other species: F  =  Ferret; D  =  Dog; R  =  Rat; S  =  Shrew; C  =  Cat; H  =  Human. For *in vivo* experiments in the rat, the table refers to the dose at which pica was observed, for all other species it refers to the emetic dose. An effect on chemotaxis (defined as a significant change in cell velocity following acute treatment (see Figure 3 and 4)) is represented by Y (Yes), with no effect denoted N (No). * Caused conditioned taste aversion responses in the rat or data derived from *in vitro* studies.

## Results

**Figure 1:**
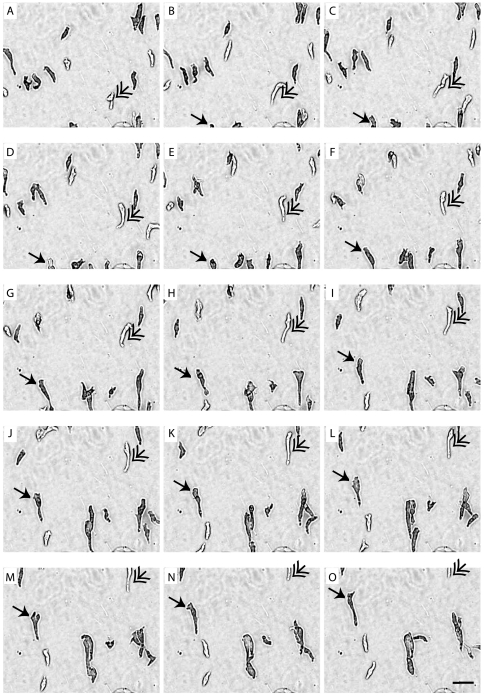
Time-dependent image series of *Dictyostelium* cells during chemotaxis. A–O: Cells moving towards a gradient of cAMP (5 µM) over a 15 min period, with images shown for each min. Two cells are indicated (arrows) over the test period. Bar  = 10 µm.

**Figure 2:**
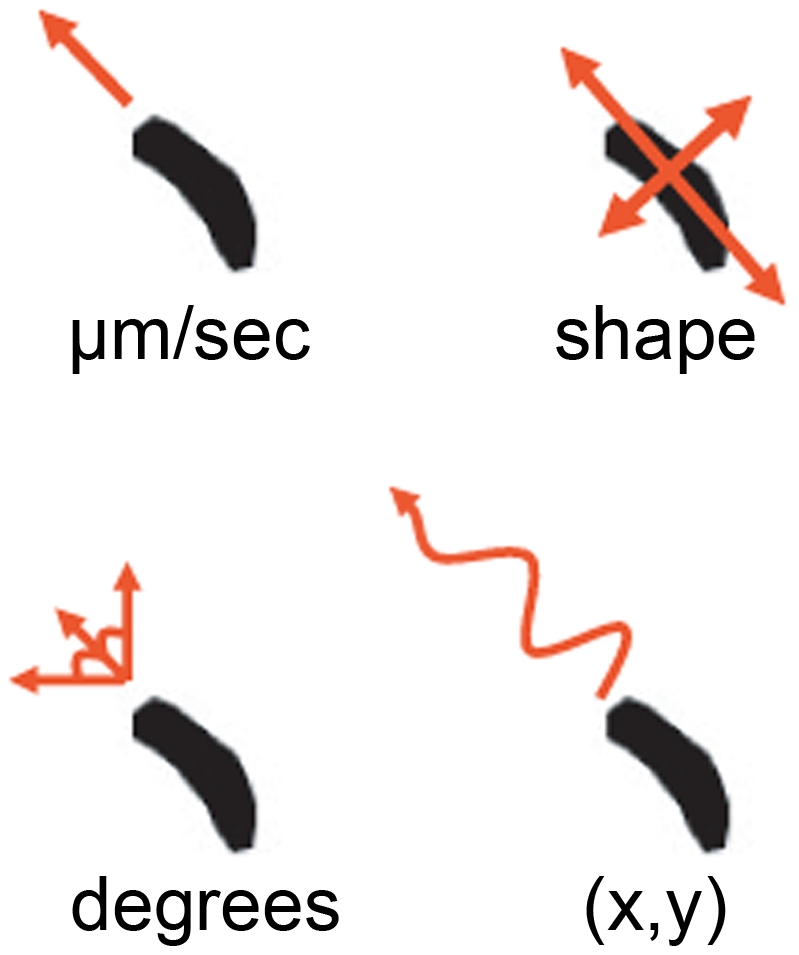
Analysis of *Dictyostelium* cell behaviour. Cells moving under a chemotactic gradient were analysed using ImageProPlus software to determine cell velocity ( µm/min); cell aspect (shape -measured as a ratio between the diameters of cells across each axis, where a value of 1 represents a circle); cell angle (degrees-where cell migration was measured in comparison to the y-axis); and cell tracking (where the co-ordinates of individual cells were illustrated following normalisation to (0,0) at 5 min) in order to illustrate changes in migration before and after compound addition.

**Figure 3:**
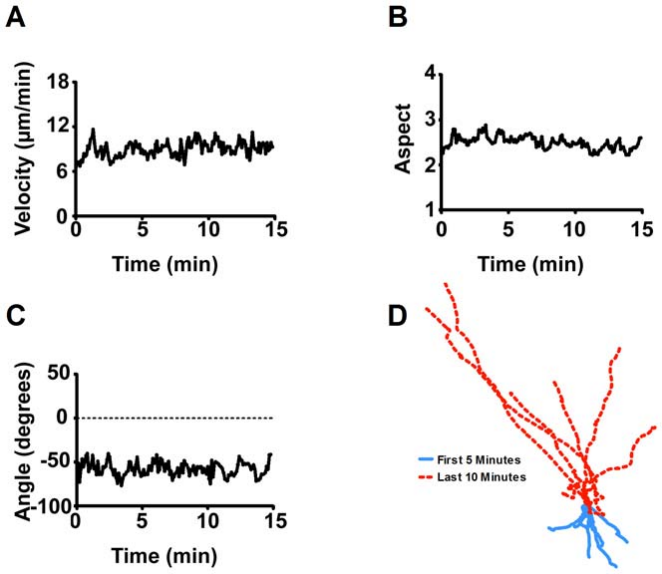
Analysis of *Dictyostelium* cell behaviour over a 15 min period under control conditions. Mean data representing 85 cell chemotaxis over a 15 min period for **A:** cell velocity; **B:** cell aspect; **C:** cell angular movement; **D:** cell tracking (where the co-ordinates of individual cells were illustrated following normalisation to (0,0) at 5 min, represented by single lines for 0–5 min (blue) or 5–15 min (dashed red) and cell direction has been adjusted so that cells are moving up the page). Data from A–C is presented as mean of triplicate experiments analysing approximately 30 cells in each.

To investigate the utility of employing *Dictyostelium* as a model for the study of tastants, cytotoxic agents, receptor agonists/antagonists and other emetic or aversive compounds, we first defined a standard assay. In this assay, *Dictyostelium* cell behaviour was monitored by time lapse photography every 6 seconds over a 15 min period (under control conditions) **(** **Figure 1** **)** within a chemotactic gradient (moving towards cAMP). Computer-generated outlines of individual cells enabled the quantification of cell velocity, aspect and angle of movement **(** **Figure 2** **and Movie S1)**. These three measurements encapsulate the complete basic behaviour of moving cells. In addition, an X, Y coordinate plot is provided illustrating the path length and direction of movement of individual cells throughout the recorded period. Under these conditions, cells exhibited stable behaviour that did not significantly change over the 15 min period monitored **(** **Figure 3** **)**.

This standard assay enabled the analysis of compounds with known emetic or aversive responses in a range of species, on *Dictyostelium* cell behaviour **(** **Table 1** **)**. For each compound and concentration, at least triplicate experiments were recorded (monitoring approximately 30 cells each), establishing the behaviour of cells for five min prior to compound addition. Following drug addition, images were then recorded for a further ten min to monitor acute drug effects. The concentrations of compounds used in these tests are based upon concentrations used *in vivo* (e.g. copper sulphate), plasma concentrations (e.g. cisplatin) or concentrations shown to be active *in vitro* in mammalian tissues relevant to the emetic reflex (e.g. RTX on neurones, denatonium on intestinal epithelial cells) as shown in **Table 1**. A compound was determined to have an effect on cell behaviour if the average cell velocity or aspect changed significantly (P<0.05) between the first five min period (prior to addition of the drug) and the final five min of the assay. Where a substance was without apparent effect at *in vitro* concentrations, experiments were then repeated at 10 – 200 fold higher concentration **(** **Table 1** **)** to reduce the risk of obtaining a false-negative result.

**Figure 4:**
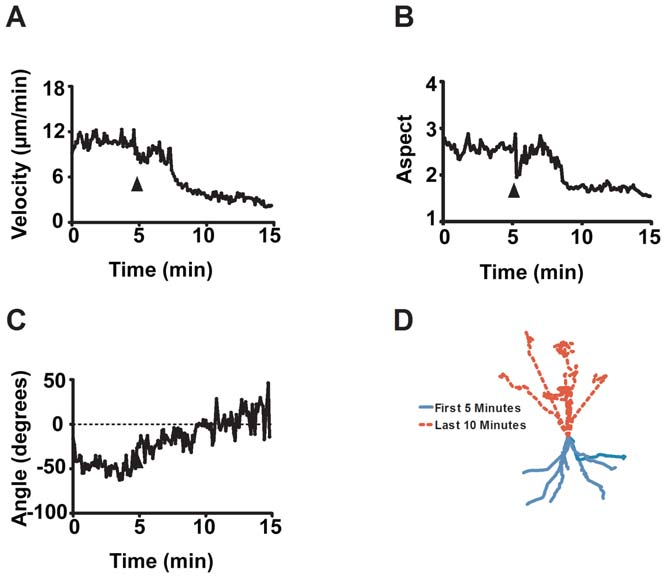
Analysis of *Dictyostelium* cell behaviour with addition of 5 mM copper sulphate after 5 Min. Mean data representing 124 cell chemotaxis over a 15 min period following addition of copper sulphate (5 mM) at 5 min (arrow) for **A:** cell velocity; **B:** cell aspect; **C:** cell angular movement; **D:** cell tracking (where the co-ordinates of individual cells were illustrated following normalisation to (0,0) at 5 min, represented by single lines for 0–5 min (blue) or 5–15 min (dashed red) and cell direction has been adjusted so that cells are moving up the page). Data from A–C is presented as a mean of triplicate experiments analysing approximately 40 cells in each.

**Figure 5:**
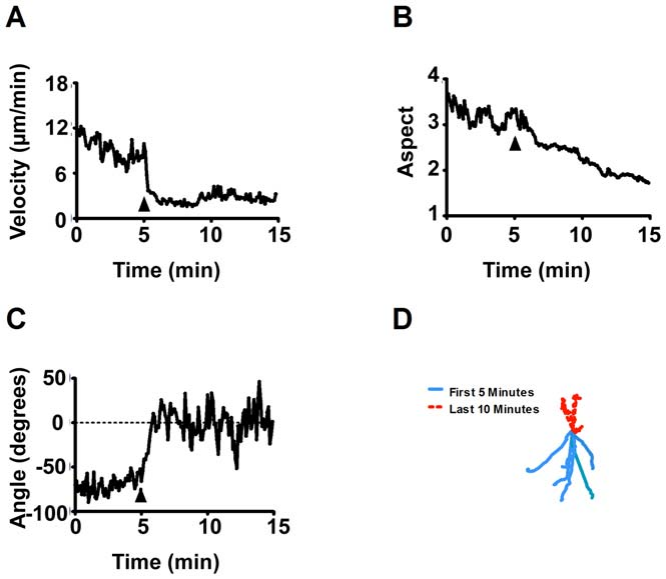
Analysis of *Dictyostelium* cell behaviour with addition of 5 mM denatonium benzoate after 5 Min. Mean data representing 89 cells chemotaxis over a 15 min period following addition of denatonium benzoate (5 mM) at 5 min (arrow) for **A:** cell velocity; **B:** cell aspect; **C:** cell angular movement; **D:** cell tracking (where the co-ordinates of individual cells were illustrated following normalisation to (0,0) at 5 min, represented by single lines for 0–5 min (blue) or 5–15 min (dashed red) and cell direction has been adjusted so that cells are moving up the page). Data from A–C is presented as a mean of triplicate experiments analysing approximately 30 cells in each.

Of the 28 compounds screened, seven evoked a significant acute effect on *Dictyostelium* cell behaviour (**Tables 1** **and** **2** **,** **Figures 4** **and** **5**). These were: denatonium benzoate, phenylthiourea, quinine hydrocholoride; copper chloride and sulphate salts; capsaicin; and rolipram. The effect of all of these compounds was a concomitant loss of velocity, cell shape and angular movement. Strength of effect also varied, where for example, addition of 5 mM copper sulphate caused *Dictyostelium* cells to slowly stop moving and lose shape over a ten min period (**Figure 4** **and Movie S2**), although still generating a significant decrease in velocity (P = 0.014) and change in aspect (P = 0.047). In contrast, the addition of denatonium benzoate (5 mM) caused an immediate loss in cell velocity and aspect **(** **Figure 5** **)**. This variation in time of onset for drug effects is also seen in the X,Y coordinate plots for these compounds (**Figure 3D** **,** **4D** **and** **5D**). Interestingly, a number of compounds that have also been shown to have tastant activity related to those tested here did not inhibit cell behaviour in this assay (e.g. the hot compound resiniferatoxin and the bitter compound cycloheximide).

**Table 2:** Statistical significance of the concentration-dependent acute reduction in *Dictyostelium* cell velocity.

Compound	Concentration (mM)	P-value (Velocity)
Capsaicin	0.01	NS
	0.05	0.025
	0.10	0.041
	0.20	0.001
	0.30	0.006
Copper Chloride	1.60	0.045
Copper Sulphate	0.16	NS
	0.80	NS
	1.20	NS
	1.60	0.032
	2.40	0.028
	5.00	0.005
Denatonium Benzoate	0.05	NS
	0.50	0.015
	1.00	0.037
	5.00	0.021
	10.0	0.06
Phenylthiourea	0.05	NS
	0.20	NS
	0.50	NS
	1.00	0.024
	2.00	0.011
	5.00	0.040
Quinine Hydrocholoride	0.05	NS
	0.10	0.049
	0.20	0.002
	0.50	0.029
	1.00	0.035
Rolipram	0.01	NS
	0.70	0.007

Concentration range of compounds showing a significant acute effect on *Dictyostelium* cell velocity between the first 5 and final 5 min of the assay. T-tests performed were 2-tailed paired student t-tests, with ∼30 cells measured in each replicate. NS  =  not significant.

**Figure 6:**
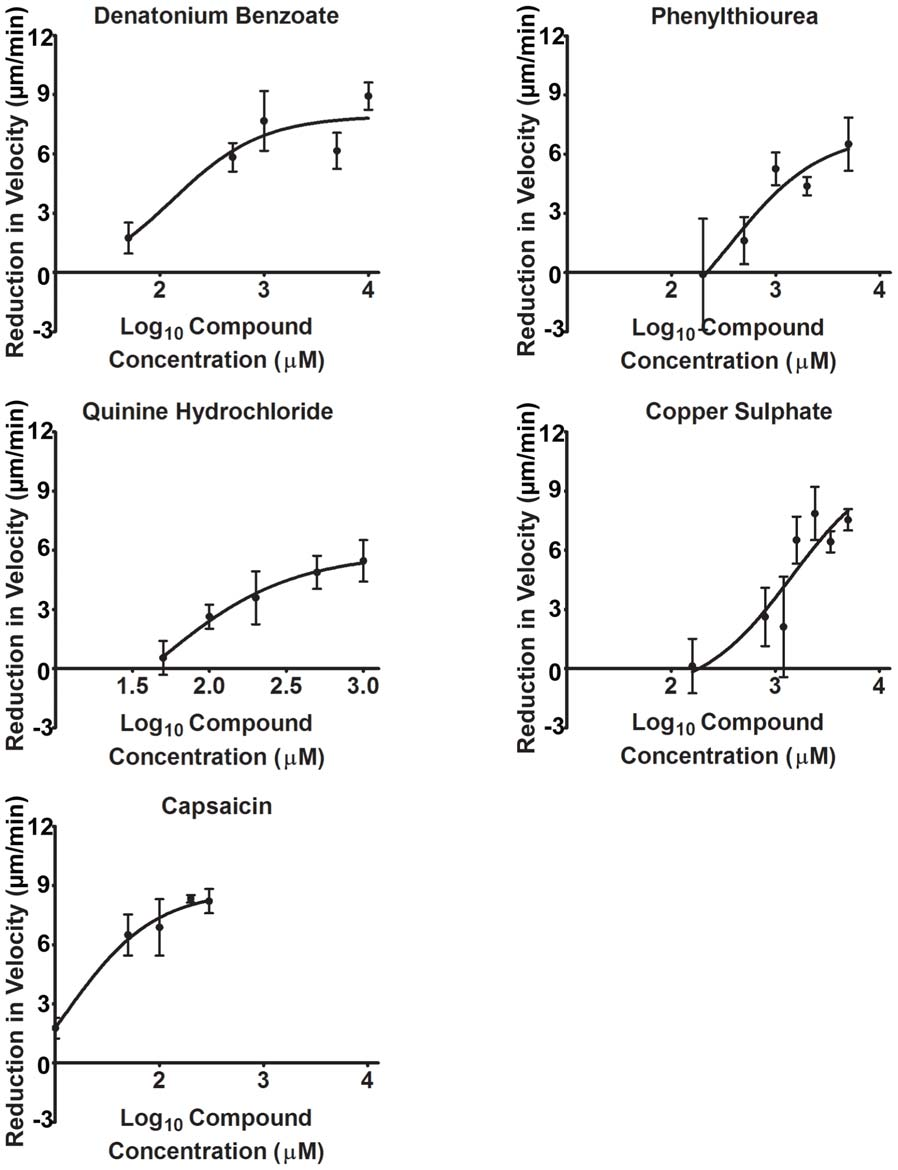
Concentration-dependent reduction of cell velocity for emetic and taste aversive compounds. Reduction in cell velocity compared to untreated cells was plotted against log_10_ concentrations, where compound concentration was chosen from those showing a non-significant effect on cell behaviour; up to a maximum of a 200-fold increase from this value. All data is presented as a mean ± S.E.M of triplicate experiments, comparing mean cell velocity during the first 5 min and final 5 min with increasing concentration.

We then investigated the concentration-dependence of this effect for denatonium benzoate, phenylthiourea, quinine hydrochloride, copper sulphate and capsaicin. A relationship between compound concentration and change in velocity was found in all cases, as indicated by the secondary plots (**Figure 6**). IC_50_ values calculated from these experiments suggest the ranking of potency to be; capsaicin (IC_50_ = 11.9±4.0 µM, R^2^ = 0.78) > quinine hydrochloride (IC_50_ = 44.3±6.8 µM, R^2^ = 0.61) > denatonium benzoate (IC_50_ = 129±4 µM, R^2^ = 0.65) > phenylthiourea (IC_50_ = 366±5 µM, R^2^ = 0.50) > copper sulphate (IC_50_ = 1433±3 µM, R^2^ = 0.54).

**Figure 7:**
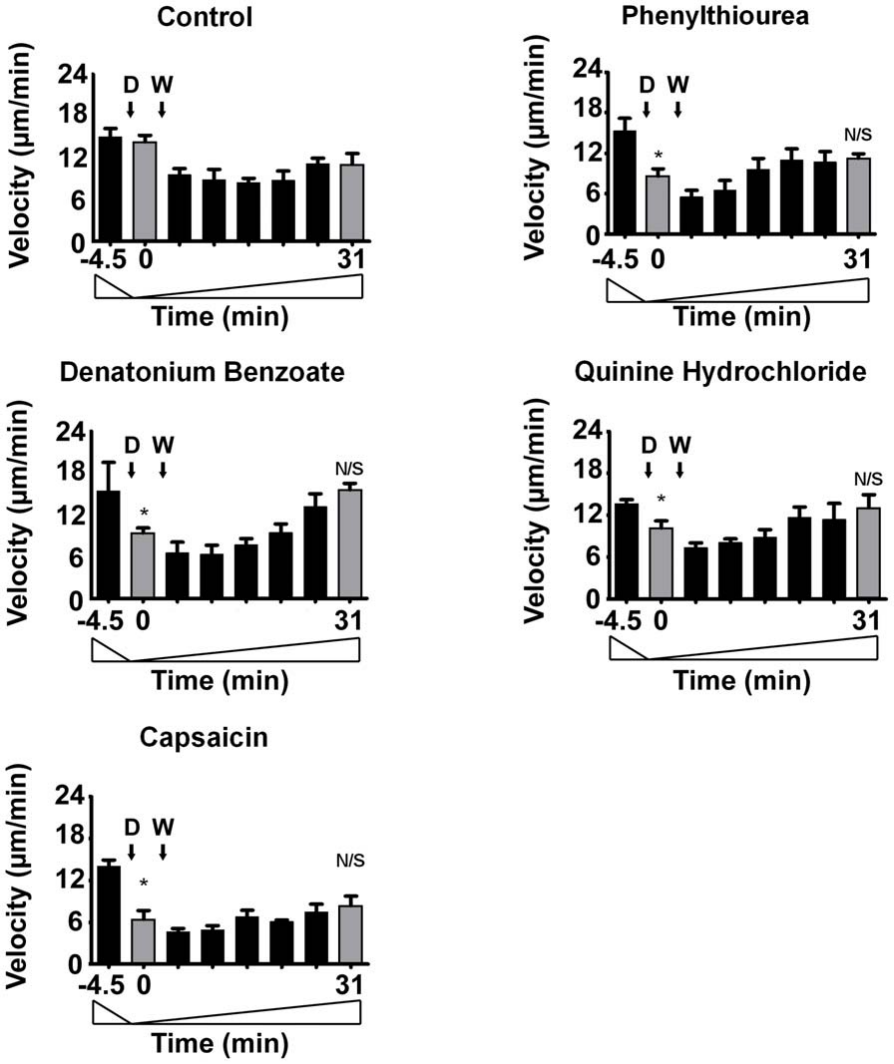
Analysis of *Dictyostelium* cell behaviour recovery post-tastant exposure. Mean data representing cell velocity during random cell movement over a 35 min period, with average cell velocity measured for 4.5 min under control conditions (at −4.5–0 min), prior to the addition of tastants at 0 min (D), followed by removal of tastants at 4.5 min (W), and the recording of recovery up to 31 min. Tastant concentrations used are: 1 mM denatonium benzoate, 3 mM phenylthiourea, 350 µM quinine hydrocholide, and 50 µM capsaicin. Data is presented as a mean ± S.E.M for each 4.5 min period, with triplicate experiments analysing approximately 30 cells in each. Grey bars indicate the equivalent time periods used in the analysis of cell velocity comparing control and each condition. N/S  =  non significant, *  =  P<0.05.

Since the block in cell behaviour during these experiments may occur through a variety of mechanisms including cell toxicity or death, we continued the analysis of tastants on *Dictyostelium* by monitoring the reversibility of behaviour effects, a potential role of cell death in this effect, and the results of long-term exposure (during development). To assess the reversibility of tastant action, we analysed the recovery of cells following compound exposure. In these experiments, cell movement was recorded for 4.5 min in the absence of a chemotactic (cAMP) gradient, prior to the addition of each tastant for 4.5 min (using concentrations at eight-fold higher that the IC_50_ value, representing approximate concentrations at which the concentration-dependence curve begins to plateau **(** **Figure 6** **)**). This length of exposure was chosen to show a significant reduction in cell velocity. The buffer containing the tastant was then replaced with fresh buffer (lacking tastant) and cells were observed for a further 26 min **(** **Figure 7** **)**. Under control conditions (in the absence of tastant), cell handling gave rise to a small non-significant drop in velocity, which then returned towards the initial velocity by the end of the test period. In the presence of all tastants, cells show an initial significant reduction in velocity upon exposure compared to untreated cells, which is consistent with earlier experiments **(** **Figure 6** **)**. However, following the removal of the 3 mM phenylthiourea, 1 mM denatonium benzoate, or 350 µM quinine hydrochloride, cells then increased in velocity, returning towards the rate of movement of untreated cells under control conditions, and showing no significant difference for the last time periods measured **(** **Figure 7** **)**. In contrast, cells exposed to 100 µM capsaicin did not recover velocity in the time period measured here (not shown). However, reducing the concentration of capsaicin to 50 µM enabled cells to recover velocity **(** **Figure 7** **)**.

To further analyse cell viability following tastant exposure, we then measured cell survival following 10 and 30 min treatment. These experiments also initially employed tastant concentrations at eight-fold IC_50_ values **(** **Figure 6** **)** and cell viability was determined using trypan blue staining **(** **Table 3** **)**. In the presence of phenylthiourea, denatonium benzoate and quinine hydrochloride, the percentage of surviving cells were above 95% after 10 min of exposure and above 90% after 30 min of exposure, indicating cell death is not the cause of the block in cell behaviour following treatment with these compounds. However, 100 µM capsaicin reduced cell survival to 51% and 57% after 10 and 30 min respectively. We therefore reduced capsaicin concentration to 50 µM, to show a 98% cells survived after both 10 and 30 min of exposure, also indicating that cell death was not the cause of capsaicin-induced block in cell movement at or below this concentration.

**Table 3:** Analysis of tastants on *Dictyostelium* cell viability.

Compound/Exposure	Mean Cell Count (alive)	S.E.M	Mean Cell Count (dead)	S.E.M	Cell Total	Cell Viability (% cells Surviving)	P-value
Time (Min)							
**Control**							
10	135	2.2	1	0.6	136	**99**	N/A
30	135	4.3	0	0	135	**100**	N/A
**1 mM DB**							
10	181	16.5	7	0	188	**96**	0.006
30	183	5.9	16	3.2	199	**92**	0.021
**350** µ**M QHCl**							
10	139	12.1	1	1	140	**99**	NS
30	146	2.59	3	1.2	150	**97**	NS
**3 mM PTU**							
10	128	8.7	0	0	128	**100**	NS
30	119	7.1	4	0.9	123	**97**	0.021
**100** µ**M Capsaicin**							
10	87	1.5	83	7.7	170	**51**	0.002
30	74	4.1	57	1.8	131	**57**	0.0004
**50** µ**M Capsaicin**							
10	174	1.3	3	1.5	177	**98**	NS
30	150	8.9	3	0.8	153	**98**	0.021

Chemotactically competent *Dictyostelium* cells were exposed to compounds at indicated concentrations and cell viability assessed after 10 and 30 min using trypan blue. All experiments were performed in triplicate. N/A  =  Not applicable NS  =  Not significant.

**Figure 8:**
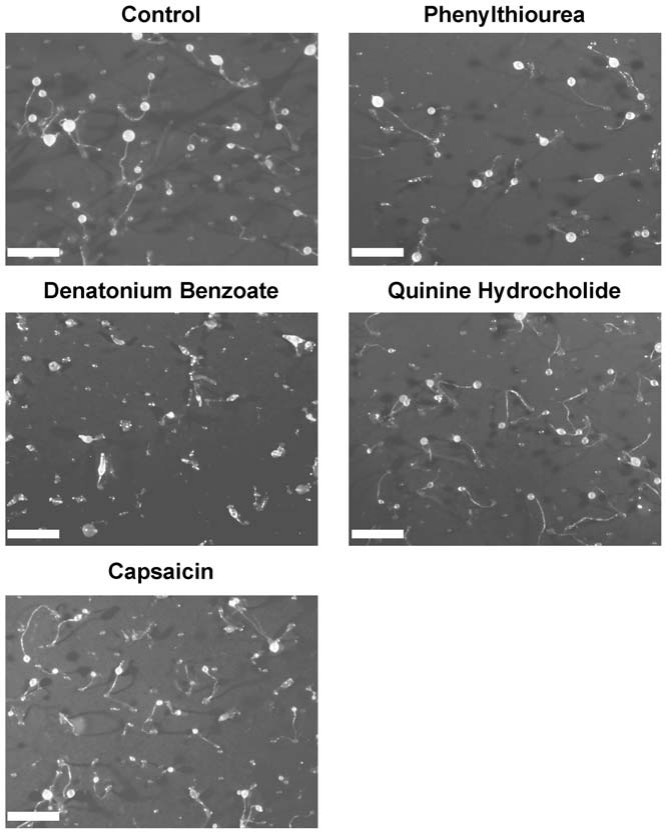
Analysis of tastants on *Dictyostelium* development. Cells were allowed to develop over 24 hours in the presence of control conditions, 3 mM phenylthiourea, 1 mM denatonium benzoate, 350 µM quinine hydrochloride and 100 µM capsaicin. All images are representative of triplicate experiments indicating cell survival after 24-hours exposure to each compound. Scale bar represents 1 mm.

Finally, we examined the chronic effect of tastants on *Dictyostelium* development by exposing cells to each compound for 24 hours during starvation on a nitrocellulose filter **(** **Figure 8** **)**. Under control conditions, cells were able to chemotax together to form a mound and ultimately develop into a multicellular fruiting body composed of a spore head held above the substratum by dead, vacuolated stalk cells [13]. Repeating these experiments in the presence of tastants (at eight-fold IC_50_ values, **Figure 6**) did not inhibit fruiting body formation **(** **Figure 8** **)**. Furthermore, development in the presence of phenylthiourea (3 mM), quinine hydrochloride (350 µM) and capsaicin (100 µM) did not alter the general structure of the fruiting body (spore head and stalk), however in the presence of denatonium benzoate (1 mM), development was slowed, with a reduced number of immature fruiting bodies present after 24 hours. This effect was overcome after prolonged incubation (48 hours; data not shown).

## Discussion

Research into the mechanisms by which diverse compounds induce emesis, and the related phenomena of pica and CTA/CFA, traditionally employs a range of unpleasant experiments on several animal species (primarily ferret, dog and rat), with considerable heterogeneity in dose and response for many compounds between different models [5]. It would thus be of significant advantage to provide early indicators of potential emetic liability during drug development prior to *in vivo* animal studies. We therefore tested a broad spectrum of emetic and aversive compounds from each major class of emesis-inducing chemical group, for their acute effects on *Dictyostelium* behaviour during chemotaxis. It was found that a range of structurally discrete tastants (capsaicin – the pungent compound in chilli peppers; and denatonium benzoate, phenylthiourea, and quinine hydrochloride – all bitter tasting compounds), a stomach irritant (copper containing compounds), and a phosphodiesterase IV (PDE4) inhibitor (rolipram) (**Table 1** **and** **2**) all caused rapid disruption of cellular behaviour, including a simultaneous decrease in cell velocity and loss of cell shape leading to rounding. Following a block in cell movement, the angle of movement also approached zero degrees. Interestingly, no treatments were found to cause changes in cell direction independent of speed or cell shape. It is worth noting that a range of emetic/aversive compounds that have been previously shown to cause chronic effects on *Dictyostelium* development, including lithium and cytotoxic compounds such as cisplatin [20], [22], [24], [25], did not acutely affect cell velocity, shape or angular direction in this study. The differences in compound effects may be due to the short exposure time (ten min) used here, in comparison to previous reports that employ treatment times of 1–24 hours.

Capsaicin, a vanilloid, is the active compound found in chillies responsible for causing a burning taste sensation and it has been demonstrated to be an agonist for the transient receptor potential vanilloid-1 (TRPV1) receptor [26]. The TRPV1 receptor has been implicated in the induction of emesis via the acute release of endogenous substance P [27], [28]. Identification of the mechanism of action of Capsaicin in *Dictyostelium* may provide a novel model for molecular research in this area, and provide further insight into a mechanism of action (through either receptor activation or inhibition, or via intracellular cell signalling pathway regulation). Initial examination of the *Dictyostelium* proteome failed to find any proteins with significant homology to known human, mouse or worm (*C. elegans*) TRPV1 proteins (based upon BLAST analysis with TRPV receptors - **Table S1**), and this therefore raises the possibility of an alternative mechanism of action of pungent tasting compounds in regulating *Dictyostelium* cell behaviour. Surprisingly, the ultra potent analogue of capsaicin, resiniferatoxin, did not significantly affect *Dictyostelium* cell behaviour at the concentrations used here, although it is capable of inducing emesis in animal models [28], [29]. It is unclear if structural or physicochemical differences between the two compounds give rise to altered efficacy in regulating the *Dictyostelium* target.

Bitter taste has been thought to be perceived in humans since a pre-Neanderthal age, providing a mechanism for identifying potentially toxic substances [30]. It has been shown that bitter taste can initiate the sensation of nausea [31]. Response to bitter tasting compounds has also been shown across a wide spectrum of model systems from mammals to frogs, fish, *Drosophila*, and *C. elegans* [32]–[38]. Denatonium benzoate, phenylthiourea and quinine hydrochloride are all bitter compounds, and all caused significant and dramatic changes in cell velocity and aspect. All three compounds are proposed T2R receptor ligands involved in this bitter taste detection, and this mechanism functions in emesis in humans at high concentrations [39]–[42]. The common effect observed here for three structurally-independent bitter compounds, at concentrations used in emetic based research **(** **Table 1** **)**, suggests a T2R receptor-like mechanism of action in *Dictyostelium*. However, BLAST analysis of the *Dictyostelium* proteome using 25 different human T2R receptors (with these proteins sharing a 30–70% homology [[43]–[45]); 24 mouse [43], six insect (*Drosophila*; NCBI 36094, 117484, 38935, 117498, 117349, 117492) [34], three worm (*C. elegans*; NCBI 178326, 177117, 188314) [32], [37] and three candidate fish (zebrafish; NCBI 664690, 553134, 798975) [35], [46] bitter receptors, again did not identify any *Dictyostelium* proteins showing significant homology within its genome **(See Table S2)**. Furthermore, other related receptors such as the mammalian TRPM5 receptor (NCBI 29850), also associated with bitter taste detection [47], [48], did not have recognisable *Dictyostelium* homologues (based on BLAST analysis – **see Table S3)**. Although the lack of recognised bitter receptors in *Dictyostelium* (as defined by protein sequence homology to other species) suggests a potential novel mechanism for the detection of bitter compounds, it must be noted that receptors for other taste-related compounds have been found in other models that lack homology to established tastant receptors (e.g. sweet receptors in *Drosophila* [49]). Identifying the mechanism (or molecular target) of bitter taste perception in *Dictyostelium* may thus provide a novel mechanism of action for bitter tasting compounds, leading to the subsequent analysis of this mechanism in humans.

Copper compounds are essential in the diet but at higher concentrations can causes gastrointestinal upsets, which include nausea, vomiting, cramps and diarrhoea [50], [51]. This has lead to the formation of regulatory guidelines for copper levels in drinking water [50], [51]. Analysis of the molecular mechanism of this effect in mammalian systems suggests the gastro-duodenal luminal concentration of copper sulphate is the key to emesis induction, thus implicating a gut mucosal-triggered reaction, and this is supported by neurophysiological studies [52]–[56]. However, the molecular emetic mechanism of copper in the gut remains unclear. Several studies have analysed a role for copper in *Dictyostelium* [57], and these have suggested that cells are highly resistant to copper through high cellular export [57]. The molecular mechanism of copper may be due to an inhibition of ATP-dependent ion currents, controlled by P2X receptors [58], [59].

Phosphodiesterase IV (PDE4) inhibitors are proposed for use as anti-inflammatory agents (e.g. in asthma) but may cause nausea and vomiting as side-effects [60]. Phosphodiesterases are responsible for degrading cAMP and cGMP in *Dictyostelium* [61], and there is one potential PDE4 homologue in *Dictyostelium* that shares a 33–35% identity with the 4 human isoforms A–D. Inhibition of phosphodiesterases would be expected to elevate extracellular cAMP levels, leading to saturation of cAMP receptors in chemotaxis, and thus the observed inhibition of cell behaviour shown in our assay. Rolipram did not exert an effect on *Dictyostelium* cell behaviour at low concentrations (10 µM), but blocked cell movement at higher concentrations (700 µM). The high concentration required for this effect may thus reflect a non-specific action of Rolipram on *Dictyostelium* phosphodiesterases [62].

The acute block in *Dictyostelium* cell behaviour caused by tastants may occur through a range of mechanisms including interaction with unknown receptor(s) or other molecular target(s), or through basic cytotoxicological mechanisms. To investigate these potential toxicological mechanisms, we carried out a range of short- and long-term exposure experiments on *Dictyostelium*. Using a concentration derived from the IC_50_ value for each compound (eight fold higher), we initially showed that all cells treated with bitter compounds recovered from acute (4.5 min) exposure **(** **Figure 7** **)**. This suggests that inhibition of cell behaviour by these tastants is not through a cytotoxicological mechanism. This was then confirmed by measuring cell death following 10 and 30 min exposure, since these compounds had no significant effect on cell viability **(** **Table 3** **)**. In contrast 100 µM capsaicin treatment did not enable cell recovery over 26 min, and caused a large reduction in cell viability following 10 min exposure. This effect however is likely to be maximal, since increased exposure (30 min) to capsaicin did not further reduce cell viability. However, reduction of capsaicin concentration to 4-fold over IC_50_ values enabled cells to recover velocity and did not reduce cell viability. These combined experiments suggest that tastants examined here do not function through an acute toxicological mechanism to block *Dictyostelium* cell behaviour.

To extend these toxicological assays for the analysis of longer exposure periods, we also examined the role of tastants on *Dictyostelium* development **(** **Figure 8** **)**. In these experiments, cells were exposed to tastants (again at eight-fold IC_50_ values) for 24 hours, and the ability to develop into mature fruiting bodies was monitored as previously described [10]–[12]. Long term exposure to all compounds did not block fruiting body formation, clearly indicating that these compounds are not lethal to *Dictyostelium* at high concentrations for extended exposure. Phenylthiourea, quinine and capsaicin exposure also did not alter fruiting body morphology, whereas denatonium benzoate slowed development, with immature fruiting bodies present after 24 hours, that later developed into mature structures. These combined cytotoxicological and developmental experiments suggest that bitter and hot compounds do not block *Dictyostelium* behaviour through toxic or irreversible mechanisms.

All pharmacological studies must consider drug concentrations, to differentiate between potential target-specific and non-specific effects. In mammalian experimental systems, bitter tasting compounds have been shown to cause effects on mouse intestinal STC-1 [63], [64] as well transfected HEK-293 cells [65] at similar concentrations to those used in our experiments (up to 10 mM and 1 mM respectively), suggesting that *Dictyostelium* is as sensitive as other models for detecting bitter taste. Similarly, capsaicin has also been used in human-based taste experiments at concentrations shown here to affect *Dictyostelium* behaviour [66], [67] (100 µM).

Since the molecular mechanisms of these compounds in chemotactic cell behaviour remains unknown, it is not possible to infer a commonly targeted signalling pathway. However, in mammalian systems, T2R receptor signalling is regulated by a TRPV1-like receptor, TRPM5, thus both ‘bitter’ and ‘hot’ compounds share a common signalling pathway [68]. Although *Dictyostelium* does not contain proteins with high amino acid sequence similarity to either of these receptors, further investigation will be necessary to determine if *Dictyostelium's* ability to detect these compounds involves a common signalling pathway.

An important limitation of this work is that primary assay used in this investigation only monitors the acute effects of test substances (within 10 min of exposure), thus any delayed effect would not be observed. For example, cytotoxic agents (e.g. cisplatin) used in anti-cancer treatments cause DNA damage, and this effect may not lead to significant changes in cell behaviour within the ten min response time recorded. These compounds do, however, give rise to a chronic block in development following longer exposure [22].

### Conclusions

A broad range of emetic and aversive compounds within the categories of tastants (e.g. bitter and hot compounds), cytotoxic agents, or generalised receptor agonists/antagonists were tested to determine if *Dictyostelium* cell behaviour could be used to investigate the molecular mechanisms of these compounds. We show that *Dictyostelium* provides a limited model for emetic or aversive compound identification. However, *Dictyostelium* may enable an exciting new avenue for research into the molecular mechanisms of bitter and hot compounds, since these compounds have a rapid and strong effect on behaviour, the compounds have an uncharacterised molecular mechanism of action, and we have demonstrated the compounds are unlikely to affect cell behaviour via toxicological means. Further investigation into the molecular mechanism of tastants on *Dictyostelium* may thus provide novel mechanism(s) of bitter and hot compound action.

## Materials and Methods

### Chemicals

The following chemicals were obtained from Sigma Aldrich Co. Ltd (Dorset, UK), and are provided with catalogue numbers: 5-fluorouracil (2,4-Dihydroxy-5-fluoropyrimidine; F6627), actinomycin D (2-Amino-(N,N)-1-bis(hexadecahydro-6,13-diisopropyl-2, 5, 9-trimethyl-1,4,7,11,14-pentaoxo-1H-pyrrolo[2], [1]-[1], [4], [7], [10], [13] oxatetraazacyclohexadecin-10-yl)-4,6-dimethyl-3-oxo-3H-phenoxazine-1,9-dicarboxamide; A1410), capsaicin (8-Methyl-N-vanillyl-*trans*-6-nonenamide; M2028), cisplatin (*cis*-Dichlorodiammine platinum(II); 479306), copper chloride (203149), copper sulphate (cupric sulphate pentahydrate; C8027), cycloheximide (3-[2-(3,5-Dimethyl-2-oxocyclohexyl)-2-hydroxyethyl] glutarimide; C7698), denatonium benzoate (*N*,*N*-Diethyl-*N*-[(2,6-dimethylphenyl carbamoyl) methyl] benzyl ammonium benzoate; D5765), digoxin (12β-Hydroxydigitoxin; D6003), fluoxetine ((±)-N-Methyl-γ-[4-(trifluoromethyl)phenoxy]benzenepropanamine hydrochloride; F132), lithium chloride (L9650), loperamide hydrochloride (4-(p-Chlorophenyl)-4-hydroxy-N,N-dimethyl-α,α-diphenyl-1-piperidinebutyramide hydrochloride; L4762), metformin (1,1-Dimethylbiguanide hydrochloride; 04635), methotrexate (4-Amino-10-methylfolic acid hydrate), nicotine (3-(1-methylpyrrolidin-2-yl)pyridine; M4010), PGF2α ((5Z,9α,11α,13E,15S)-9,11,15-Trihydroxyprosta-5,13-dienoic acid tris salt; P0424), phenylthiourea (1-Phenyl-2-thiourea; P7629), pyrogallol (1,2,3-Trihydroxybenzene; P0381), quinine hydrochloride ((R)-[(2S,4R,5R)-5- ethenyl-1 -azabicyclo [2.2.2]octan-2-yl]- (6-methoxyquinolin-4-yl) methanol dihydrate hydrochloride; Q1125), resiniferatoxin (4-Hydroxy-3-methoxy- [(2S,3aR,3bS,6aR,9aR,9bR,10R,11aR)- 3a,3b,6,6a,9a, 10,11,11a-octahydro-6a-hydroxy-8, 10-dimethyl-11a-(1-methylethenyl)-7-oxo-2-(phenylmethyl)-7H-2,9b -epoxyazuleno[5,4-e]-1,3-benzodioxol-5-yl] benzeneacetate; R8756), rolipram (4-[3-(Cyclopentyloxy)-4-methoxyphenyl]-2-pyrrolidinone; R6520), streptozocin (*N*-(Methylnitrosocarbamoyl)-α-D-glucosamine; S0130), veratridine (3-Veratroylveracevine; V5754), vincristine (22-Oxovincaleukoblastine sulfate salt; V8388) and zinc sulphate monohydrate (96495). The following compound (with catalogue number) were obtained from Tocris Bioscience Ltd: (Bristol, UK) 5-hydroxytryptamine (3-(2-Aminoethyl)-1H-indol-5-ol hydrochloride; 3547), apomorphine hydrochloride (R(–)-10,11-Dihydroxyaporphine; 2073) and substance P ((2S)-2-[[(2S)-1-[(2S)-6-amino-2-[[(2S)-1-[(2S)-2-amino-5-(diaminomethylideneamino) pentanoyl]pyrrolidine-2-carbonyl]amino]hexanoyl]pyrrolidine-2-carbonyl]amino]-N-[(2S)-5-amino-1-[[(2S)-1-[[(2S)-1-[[2-[[(2S)-1-[[(2S)-1-amino-4-methylsulfanyl-1-oxobutan-2-yl] amino]-4-methyl-1-oxopentan-2-yl]amino]-2-oxoethyl]amino]-1-oxo-3-phenylpropan-2-yl] amino]-1-oxo-3sphenylpropan-2-yl]amino]-1,5-dioxopentan-2-yl]pentanediamide; 1156). All compounds were dissolved in dimethylsulfoxide (DMSO) (5-fluorouracil, actinomycin D, cycloheximide, digoxin, fluoxetine, rolipram, quinine hydrochloride, capsaicin and resiniferatoxin) or phosphate buffer (16.5 mM KH_2_PO_4_, 3.8 mM K_2_HPO_4_, pH 6.2) (lithium chloride, cisplatin, streptozotocin, vincristine, metformin, pyrogallol, copper sulphate, copper chloride, zinc sulphate, substance P, nicotine, loperamide hydrochloride, PGF_2α_, denatonium benzoate, phenylthiourea), apart from 5-hydroxytryptamine, apomorphine hydrochloride (dissolved in 0.9% ascorbic acid); methotrexate and veratridine (dissolved in 0.1% sodium hydroxide).

### Cell Behaviour Assay

To prepare *Dictyostelium* cells (Ax2) for behaviour analysis experiments, cells were grown in shaking suspension in Axenic medium (Formedium Co. Ltd, Norfolk, UK), washed and resuspended in phosphate buffer at 1.7×10^6^ cells/ml. Cells were then pulsed for 5 hours with 30 nΜ cyclic adenosine monophosphate (cAMP) (Sigma Co. Ltd, Dorset, UK) at 6 min intervals whilst shaking at 120 rpm. Cells were then washed in phosphate buffer, resuspended at 1×10^7^ cells/ml, and used in a Dunn chamber (Hawksley, Sussex, UK) assay [69], migrating toward 5 µM cAMP. A stable chemotactic gradient was allowed to form over a 30 min period, prior to recording cell shape and position using an Olympus IX71 microscope at 40x magnification with a QImaging RetigaExi Fast1394 digital camera. Cell images were recorded every 6 seconds over a 15 min period, with the initial 5 min period recorded prior to addition of test compounds (within a 10 µL aliquot diluted in 5 µM cAMP) to the outer well of the Dunn chamber. Subsequent images were recorded over the following ten min period for each compound, and at each concentration, with a minimum of three independent experiments for each drug/concentration and an average cell number of ∼30 cells quantified per experiment. Cell recordings were prepared in the second quadrant of the Dunn chamber, enabling cell angular movement to be recorder at around −50 degrees. Solvent only controls were carried out for all experiments to ensure readouts were based upon compounds listed, with for example, no effect of DMSO shown at 0.6%– the highest concentration used in the experiments described here.

### *Dictyostelium* Recovery Following Tastant Exposure

*Dictyostelium* cells (Ax2) were pulsed as described above, re-suspended at 1.7×10^5^ cells/mL, and 250 µL aliquots of cells were added to Lab-Tek 8-well chambered coverglass wells (Thermo Fisher, Leicestershire, UK) and allowed to adhere for 45 min. Cell movement was recorded as above at intervals of 18 seconds for a total of 35 min (in the absence of a chemotactic gradient). Cells were allowed to establish a base-line velocity for 4.5 min, prior to the addition of 10 µL of tastant to give indicated final concentrations. Following 4.5 min tastant exposure, cell buffer containing tastants was aspirated from the chamber and replaced with 250 µL phosphate buffer (over a 30 second period), and cells were monitored for a further 25.5 min. All experiments were performed in a minimum of triplicate individual assays, at each compound concentration.

### Cell Viability Assay

*Dictyostelium* cells (Ax2) were pulsed as described, re-suspended at 2.5×10^5^ cells/mL, exposed to tastants (at indicated concentrations) for 7 or 27 min and then stained with 0.4% trypan blue solution (final concentration 0.067%) for 3 min prior to live counting. Dead cells were identified as a distinctive blue colour since live cells did not change colour. Experiments were performed in triplicate.

### Development Assay

*Dictyostelium* development assays were performed in triplicate experiments as previously described [13], [70], [71].

### Data Analysis and Statistics

Changes in cell velocity, aspect (the ratio between the major and minor axes of an elliptical shape such as a cell) and angular movement **(** **Figure 2** **)** were monitored for every cell within each of the 600 frames recorded over the 15 min period and analysed by ImagePro software (Media Cybernetics, Buckinghamshire, UK). Compound effects were compared using the mean velocity and aspect of cells between the first 5 min and the final 5 min and significance was determined using a two-tailed paired student t-test (P≤0.05). Angle was also measured throughout in order to observe any changes in the direction of cell movement. The relative co-ordinates of cells (X,Y) were also mapped and represented in a line-tracking plot whereby the co-ordinates of cells at 5 min (when the compound was added) were normalised to (0,0).

In quantifying velocity, concentration-related drug response was calculated by subtracting the mean velocity of cell movement in the final five min from the mean velocity in the first 5 min for each compound at each concentration. These were plotted against drug concentration (log_10_) to quantify the change in velocity with drug concentration. A non-linear three parameter log _10_ concentration response curve was fitted by GraphPad Prism (GraphPad Software Inc v5.02, San Diego, USA) using a least squares fit. Using GraphPad, the subsequent concentration at half maximal compound inhibition (IC_50_) was calculated well as the R^2^ value in order to display the accuracy of the curve fit.

Cell velocity was quantified in the *Dictyostelium* recovery experiments as described above. Significance was determined using unpaired one-tailed student t-tests (based on a unidirectional drop in velocity as observed in *Dictyostelium* cell behaviour assay) by comparing mean velocity of control cells and equivalent mean velocities at each compound concentration. Tastant dependent effects were determined by comparing 0–4.5 min and 27-31 min periods (grey bar) between control and tastant treated cells to assess initial decrease in cell velocity and recovery of velocity **(** **Figure 7** **)**.

Significance was determined in cell viability assays by comparing the percentage cell viability between control conditions and each compound concentration using paired 2-tailed student t-tests.

## Supporting Information

Movie S1 ***Dictyostelium*** **chemotaxis was monitored by time-lapse photography to record cells moving within a Dunn Chamber towards the chemo-attractant, cAMP, across the screen from left to right.** Images were taken every 6 seconds over a 15 minute period. Computer generated cell outlines enables average cell velocity, shape and direction of movement to be quantified.
(AVI)

Movie S2 ***Dictyostelium*** **chemotaxis was monitored by time-lapse photography to record cells moving within a Dunn Chamber towards the chemo-attractant, cAMP, across the screen from bottom right to top left.** Images were taken every 6 seconds over a 15 minute period. Computer generated cell outlines enables average cell velocity, shape and direction of movement to be quantified. Cell movement was recorded over a 5 minute period, prior to the addition a range of emetic or aversive compounds (indicated by a black screen flash), and cell behaviour was recorded for a further 10 minutes. In the movie shown here, the stomach irritant copper sulphate (5 mM ) was added, causing *Dictyostelium* cells to slowly stop moving and lose shape over the ten min period tested.
(AVI)

Table S1 **Homology search results (BLAST analysis) of the** ***Dictyostelium*** **genome for proteins showing amino acid similarity to TRPV receptors from multiple species.** Potential homologues are defined by an E-value of less than 1.00E-40 [11], thus *Dictyostelium* does not contain proteins showing significant sequence similarity to be considered as homologues.
(DOCX)

Table S2 **Homology search results (BLAST analysis) of the** ***Dictyostelium*** **genome for proteins showing amino acid similarity to known bitter receptors from multiple species.** Potential homologues are defined by an E-value of less than 1.00E-40 [11], thus *Dictyostelium* does not contain proteins showing significant sequence similarity to be considered as homologues. N/A  =  Not applicable.
(DOCX)

Table S3 **Homology search results (BLAST analysis) of the** ***Dictyostelium*** **genome for proteins showing amino acid similarity to TRPM5 receptors from human and mouse.** Potential homologues are defined by an E-value of less than 1.00E-40 [11], thus *Dictyostelium* does not contain proteins showing significant sequence similarity to be considered as homologues. N/A  =  Not applicable.
(DOCX)
