# Supplementary material for: Investigating the Effect of Emetic Compounds on Chemotaxis in Dictyostelium Identifies a Non-Sentient Model for Bitter and Hot Tastant Research
Source: PLoS One. 2011 Sep 8;6(9):e24439. doi: 10.1371/journal.pone.0024439 (PMC3169598; doi:10.1371/journal.pone.0024439)
Supplement: Table S1 — Homology search results (BLAST analysis) of the Dictyostelium genome for proteins showing amino acid similarity to TRPV receptors from multiple species. Potential homologues are defined by an E-value of less than 1.00E-40 [11], thus Dictyostelium does not contain proteins showing significant sequence similarity to be considered as homologues. (DOCX) [file pone.0024439.s003.docx]

| **NCBI Number** | **Gene** | **Organism** | **Number**  **of *Dictyostelium*** | **Highest** |
| --- | --- | --- | --- | --- |
|  |  |  | **BLAST Hits** | **E-value** |
| 7442 | TRPV1 | *H. Sapiens* | >10 | 0.001 |
| 51393 | TRPV2 | *H. Sapiens* | 5 | 0.0006 |
| 162514 | TRPV3 | *H. Sapiens* | 8 | 6.00E-05 |
| 59341 | TRPV4 | *H. Sapiens* | 4 | 0.002 |
| 56302 | TRPV5 | *H. Sapiens* | >10 | 5.00E-09 |
| 55503 | TRPV6 | *H. Sapiens* | 7 | 2.00E-05 |
| 193034 | TRPV1 | *M. musculus* | >10 | 4.00E-04 |
| 22368 | TRPV2 | *M. musculus* | 1 | 1.00E-04 |
| 246788 | TRPV3 | *M. musculus* | 10 | 2.00E-05 |
| 63873 | TRPV4 | *M. musculus* | 3 | 5.00E-03 |
| 194352 | TRPV5 | *M. musculus* | >10 | 5.00E-06 |
| 64177 | TRPV6 | *M. musculus* | >10 | 6.00E-09 |
| 177117 | OSM-9 | *C. elegans* | >10 | 5.00E-06 |
| 188314 | OCR-2 | *C. elegans* | >10 | 5.00E-07 |
