# Supplementary material for: Investigating the Effect of Emetic Compounds on Chemotaxis in Dictyostelium Identifies a Non-Sentient Model for Bitter and Hot Tastant Research
Source: PLoS One. 2011 Sep 8;6(9):e24439. doi: 10.1371/journal.pone.0024439 (PMC3169598; doi:10.1371/journal.pone.0024439)
Supplement: Table S2 — Homology search results (BLAST analysis) of the Dictyostelium genome for proteins showing amino acid similarity to known bitter receptors from multiple species. Potential homologues are defined by an E-value of less than 1.00E-40 [11], thus Dictyostelium does not contain proteins showing significant sequence similarity to be considered as homologues. N/A = Not applicable. (DOCX) [file pone.0024439.s004.docx]

| **NCBI Number** | **Gene** | **Organism** | **Number**  **of *Dictyostelium***  **BLAST Hits** | **Highest**  **E-value** |
| --- | --- | --- | --- | --- |
| 50834 | TAS2R1 | *H. sapien* | 0 | N/A |
| 50831 | TAS2R3 | *H. sapien* | 0 | N/A |
| 50832 | TAS2R4 | *H. sapien* | 0 | N/A |
| 54429 | TAS2R5 | *H. sapien* | 0 | N/A |
| 50837 | TAS2R7 | *H. sapien* | 0 | N/A |
| 50836 | TAS2R8 | *H. sapien* | 0 | N/A |
| 50835 | TAS2R9 | *H. sapien* | 0 | N/A |
| 50839 | TAS2R10 | *H. sapien* | 0 | N/A |
| 50838 | TAS2R13 | *H. sapien* | 0 | N/A |
| 50840 | TAS2R14 | *H. sapien* | 0 | N/A |
| 50833 | TAS2R16 | *H. sapien* | 0 | N/A |
| 259294 | TAS2R19 | *H. sapien* | 0 | N/A |
| 259295 | TAS2R20 | *H. sapien* | 0 | N/A |
| 259293 | TAS2R30 | *H. sapien* | 0 | N/A |
| 259290 | TAS2R31 | *H. sapien* | 0 | N/A |
| 5726 | TAS2R38 | *H. sapien* | 0 | N/A |
| 259285 | TAS2R39 | *H. sapien* | 0 | N/A |
| 259286 | TAS2R40 | *H. sapien* | 0 | N/A |
| 259287 | TAS2R41 | *H. sapien* | 1 | 0.034 |
| 353164 | TAS2R42 | *H. sapien* | 0 | N/A |
| 259289 | TAS2R43 | *H. sapien* | 0 | N/A |
| 259291 | TAS2R45 | *H. sapien* | 0 | N/A |
| 259292 | TAS2R46 | *H. sapien* | 0 | N/A |
| 259296 | TAS2R50 | *H. sapien* | 0 | N/A |
| 338398 | TAS2R60 | *H. sapien* | 0 | N/A |
| 387339 | TAS2R102 | *M. musculus* | 0 | N/A |
| 667992 | TAS2R103 | *M. musculus* | 0 | N/A |
| 387340 | TAS2R104 | *M. musculus* | 0 | N/A |
| 57252 | TAS2R105 | *M. musculus* | 0 | N/A |
| 387341 | TAS2R106 | *M. musculus* | 0 | N/A |
| 387342 | TAS2R107 | *M. musculus* | 0 | N/A |
| 387343 | TAS2R109 | *M. musculus* | 0 | N/A |
| 387344 | TAS2R110 | *M. musculus* | 0 | N/A |
| 387345 | TAS2R113 | *M. musculus* | 0 | N/A |
| 387346 | TAS2R114 | *M. musculus* | 0 | N/A |
| 112408 | TAS2R116 | *M. musculus* | 0 | N/A |
| 353166 | TAS2R117 | *M. musculus* | 0 | N/A |
| 57254 | TAS2R119 | *M. musculus* | 0 | N/A |
| 387348 | TAS2R120 | *M. musculus* | 0 | N/A |
| 353167 | TAS2R123 | *M. musculus* | 0 | N/A |
| 387351 | TAS2R124 | *M. musculus* | 0 | N/A |
| 387352 | TAS2R125 | *M. musculus* | 0 | N/A |
| 387353 | TAS2R126 | *M. musculus* | 0 | N/A |
| 387354 | TAS2R129 | *M. musculus* | 0 | N/A |
| 387511 | TAS2R134 | *M. musculus* | 0 | N/A |
| 387512 | TAS2R135 | *M. musculus* | 0 | N/A |
| 353165 | TAS2R136 | *M. musculus* | 0 | N/A |
| 387616 | TAS2R140 | *M. musculus* | 0 | N/A |
| 387514 | TAS2R143 | *M. musculus* | 0 | N/A |
| 664690 | TAS2R200.1 | *D.rerio* | 0 | N/A |
| 553134 | TAS2R200.2 | *D.rerio* | 0 | N/A |
| 798975 | TAS2R202 | *D.rerio* | 0 | N/A |
| 36094 | GR47A | *D. melanogastar* | 0 | N/A |
| 117484 | GR59B | *D. melanogastar* | 0 | N/A |
| 38935 | GR66A | *D. melanogastar* | 0 | N/A |
| 117498 | GR22E | *D. melanogastar* | 0 | N/A |
| 117349 | GR22F | *D. melanogastar* | 0 | N/A |
| 117492 | GR22B | *D. melanogastar* | 0 | N/A |
| 178236 | QUI-1 | *C. elegans* | >10 | 2.00E-19 |
| 177117 | OSM-9 | *C. elegans* | >10 | 5.00E-06 |
| 188314 | OCR-2 | *C. elegans* | >10 | 5.00E-07 |
