# Supplementary material for: Investigating the Effect of Emetic Compounds on Chemotaxis in Dictyostelium Identifies a Non-Sentient Model for Bitter and Hot Tastant Research
Source: PLoS One. 2011 Sep 8;6(9):e24439. doi: 10.1371/journal.pone.0024439 (PMC3169598; doi:10.1371/journal.pone.0024439)
Supplement: Table S3 — Homology search results (BLAST analysis) of the Dictyostelium genome for proteins showing amino acid similarity to TRPM5 receptors from human and mouse. Potential homologues are defined by an E-value of less than 1.00E-40 [11], thus Dictyostelium does not contain proteins showing significant sequence similarity to be considered as homologues. N/A = Not applicable. (DOCX) [file pone.0024439.s005.docx]

| **NCBI Number** | **Gene** | **Organism** | **Number**  **of *Dictyostelium***  **BLAST Hits** | **Highest**  **E-value** |
| --- | --- | --- | --- | --- |
| 29850 | TRPM5 | *H. Sapiens* | 0 | N/A |
| 56843 | TRPM5 | *M. musculus* | 0 | N/A |
